# Effects of elevated pCO_2_ and nutrient enrichment on the growth, photosynthesis, and biochemical compositions of the brown alga *Saccharina japonica* (Laminariaceae, Phaeophyta)

**DOI:** 10.7717/peerj.8040

**Published:** 2019-11-27

**Authors:** Yaoyao Chu, Yan Liu, Jingyu Li, Qingli Gong

**Affiliations:** 1 College of Fisheries, Ocean University of China, Qingdao, Shandong, China; 2 The Key Laboroatory of Mariculture (Ocean University of China), Ministry of Education, Qingdao, Shandong, China

**Keywords:** Ocean acidification, Eutrophication, *Saccharina japonica*, Growth, Photosynthesis, Biochemical compositions

## Abstract

Ocean acidification and eutrophication are two major environmental issues affecting kelp mariculture. In this study, the growth, photosynthesis, and biochemical compositions of adult sporophytes of *Saccharina japonica* were evaluated at different levels of pCO_2_ (400 and 800 µatm) and nutrients (nutrient-enriched and non-enriched seawater). The relative growth rate (RGR), net photosynthetic rate, and all tested biochemical contents (including chlorophyll (Chl) *a*, Chl *c*, soluble carbohydrates, and soluble proteins) were significantly lower at 800 µatm than at 400 µatm pCO_2_. The RGR and the contents of Chl *a* and soluble proteins were significantly higher under nutrient-enriched conditions than under non-enriched conditions. Moreover, the negative effects of the elevated pCO_2_ level on the RGR, net photosynthetic rate, Chl *c* and the soluble carbohydrates and proteins contents were synergized by the elevated nutrient availability. These results implied that increased pCO_2_could suppress the growth and biochemical composition of adult sporophytes of *S. japonica*. The interactive effects of ocean acidification and eutrophication constitute a great threat to the cultivation of *S. japonica* due to growth inhibition and a reduction in quality.

## Introduction

Due to intensive anthropogenic activities in recent years, the level of CO_2_ in the atmosphere has increased from 285 µatm in 1975 to >400 µatm at present (IPCC, 2014). This caused an increase in the concentration of dissolved CO_2_ in the ocean, resulting in a decrease in seawater pH, which is called ocean acidification (OA). Because of OA, the seawater carbonate system has also changed, affecting the physiological performances of marine organisms, species interactions, and coastal ecosystems (Connell & Russell, 2010; Enochs et al., 2015; Myers et al., 2017; Ullah et al., 2018). On the other hand, due to a large amount of wastewater emissions derived from industrial and agricultural production, eutrophication has been another important environmental issue affecting coastal waters worldwide (Geertz-Hansen et al., 1993; Smith et al., 2003). Excessive nutrient inputs can lead to harmful algal blooms (Bricker et al., 2008; Cai et al., 2011; Smetacek & Zingone, 2013), which decrease oxygen concentrations in the water column and produce toxins that might be lethal to marine organisms, thereby resulting in shifts in species dominance and community structure (Glibert et al., 2005; Xiao et al., 2017).

Kelp are the dominant taxa in the sublittoral zones of temperate and polar coastal regions worldwide and provide habitat and nursery ground for a large number of marine organisms (Steneck et al., 2002; Graham, 2004). Due to their ecological importance, many studies have focused on how environmental changes, such as OA and eutrophication, affect the growth and physiology of kelp species (Gaitán-Espitia et al., 2014; Agatsuma et al., 2014; Oh, Yu & Choi, 2015; Gao et al., 2017c). Some results showed that OA negatively influenced the microscopic development of *Macrocystis pyrifera* and *Undaria pinnatifida* (Gaitán-Espitia et al., 2014; Xu et al., 2015; Gao et al., 2019a), which may be due to the inhibition of cell activities (Ragazzola et al., 2012). In contrast, kelp sporophytes exhibited positive physiological responses to OA (Schmid, Mills & Dring, 1996; Olischlaeger et al., 2012; Gao et al., 2019a; Gao et al., 2019b) due to stimulation of photosynthetic activities by increased inorganic carbon (Olischlaeger et al., 2012). Additionally, kelp species benefit from an increase in nutrient availability in seawater. For example, a nutrient supply increased the maturation of gametophytes and the growth and production of sporophytes (Mizuta, Narumi & Yamamoto, 2001; Agatsuma et al., 2014; Endo et al., 2017). Despite the important effects of OA and eutrophication on kelp species, very few studies have considered the combined effects of these factors on the physiological characteristics of kelp species.

The canopy-forming kelp *Saccharina japonica* Areschoug inhabits subtidal zones of northwestern Pacific countries, including Japan, Korea and China (Selivanova, Zhigadlova & Hansen, 2007; Liu et al., 2009). This kelp has also been commercially cultivated in these countries because adult sporophytes are used as food for humans and as raw industrial materials (Liu et al., 2012; Liu et al., 2014; Hwang et al., 2018). Previous studies on the physiological responses of *S. japonica* to OA focused mainly on the microscopic stages (Xu et al., 2015; Gao et al., 2019a) and juvenile sporophyte (Gao et al., 2019a). However, the responses of adult sporophytes to OA should also be investigated because many seaweeds showed different responses to OA during different developmental stages (Olischlaeger et al., 2012; Gao et al., 2018a; Gao et al., 2018b). OA could also significantly affect the biochemical composition of seaweeds, including protein and carbohydrates (Andria, Vergara & Perez-Llorens, 1999; Xu et al., 2017; Gao et al., 2018a), which is considered a criterion to measure the quality of cultivated algae. On the other hand, an increase in the nutrient concentration significantly enhanced the growth, photosynthetic activities, and nutrient uptake of *S. japonica* (Mizuta & Maita, 1991; Gao et al., 2017c). Nevertheless, very few studies have been conducted to evaluate the potential interactive effect of OA and eutrophication on the growth and quality of this kelp species.

Therefore, in the present study, we investigated the combined effects of OA and eutrophication on the growth, photosynthesis, and biochemical composition of adult sporophytes of *S. japonica*. According to previous studies, we hypothesized that OA would inhibit the growth and quality of this kelp and that excessive nutrients would affect its physiological responses to OA. The results of this study are expected to provide valuable information for improving the cultivation production of *S. japonica* in China.

## Materials & Methods

### Algal collection and maintenance

Adult sporophytes of *S. japonica* were collected from cultivated populations in Rongcheng, Shandong, China (36°07′N, 120°19′E), in May 2018. The samples were transported quickly to the laboratory using a cold plastic box filled with seawater within 5 h. Healthy sporophytes were selected and rinsed several times with sterilized seawater to remove epiphytic organisms and detritus. More than 60 discs (1.4 cm in diameter) were punched from the meristem with a cork borer for the subsequent experiments. The discs were stock-cultured in a plastic tank containing 6 L filtered seawater, which was obtained from the coast of Taipingjiao, Qingdao with a salinity of approximately 30 psu. These discs were kept at an irradiance of 180 µmol photons m^−2^ s^−1^, a 12:12 h light/dark cycle, and 10 °C, which was the seawater temperature of the collection area, for 3 days to reduce the negative effects of excision.

### Culture experiment and growth

A culture experiment was conducted over a period of 6 days under combinations of two pCO_2_ levels (400 and 800 µatm) and two nutrient levels (non-enriched natural seawater and nutrient-enriched seawater). There was a total of 4 experimental treatments and each treatment had three replicates. During the experiment, a light/dark cycle of 12:12 h and an irradiance of 180 µmol photon m^−2^ s^−1^ were held constant. This experiment used 12 side-arm flasks, with each flask containing 500 mL of natural seawater or 50% PESI-enriched seawater (Tatewaki, 1966). In the natural seawater treatment, the culture medium contained 27 µM NO_3_^−^ and 2 µM H_2_PO_4_^−^, and the cultures with 50% PESI-enriched seawater contained 437 µM NO_3_^−^ and 28 µM H_2_PO_4_^−^. The elevated nutrient level was based on studies referring to eutrophication (Ménesguen et al., 2018; Gao et al., 2019c), and nutrient limiting did not occur at the applied nutrient level during the experiment based on a preliminary experiment. Four discs were put into each flask, which was then gently aerated. The culture medium was renewed every 3 days.

For the experimental treatment, two pCO_2_ levels were maintained in two CO_2_ incubators: 400 µatm (ambient air) and 800 µatm (elevated pCO_2_). The CO_2_ levels were automatically regulated in two incubators (GXZ-380C-C02, Jiangnan Instruments Factory, Ningbo, China) by controlling the flow of ambient air and pure CO_2_ gas. Autoclaved natural seawater with the present pCO_2_ level (approximately 400 µatm) was used as a control. A pH meter (Orion STAR A211; Thermo Scientific) was used to measure the pH value of the medium in each flask. Total alkalinity (TA) was measured using an automatic alkalinity titrator by Gran acidimetric titration (848MPT, Titrino). The samples used to measure the TA were poisoned with a saturated solution of mercuric chloride after filtering through cellulose acetate membranes (0.22 µm). The seawater carbonate chemistry parameters were calculated based on the values of the pH, TA, salinity, nutrients, the equilibrium constants K1 and K2 for carbonic acid dissociation (Roy et al., 1993), and KB for boric acid (Dickson, 1990), using CO2SYS software (Lewis & Wallace, 1998).

At the end of the experiment, the fresh weights of the discs were measured after being blotted with tissue paper. The relative growth rate (RGR) of each replicate was calculated using the following formula:

\begin{eqnarray*}& & \mathrm{RGR}(\text{%} {\mathrm{day}}^{-1})=100 \mathrm{ln} ({W}_{t}/{W}_{0})/t \end{eqnarray*}

where *W*_0_ is the initial fresh weight, *W*_*t*_ is the final fresh weight, and *t* is the number of days.

### Photosynthesis measurements

After the culture experiment, the net photosynthetic rate (*P*_*n*_) of the discs was obtained using a manual FireSting O_2_ II oxygen meter (Firesting O_2_, Pyro Science). After measuring the fresh weight, four discs were transferred to the oxygen electrode cuvette with 330 mL of medium from the culture flask. Then the medium was magnetically stirred during the measurement to ensure even diffusion of oxygen. The temperature and light conditions were the same as for the abovementioned culture experiment. Prior to the measurements, the samples were allowed to acclimate to the conditions in the cuvette for 5 min. The oxygen concentration in the medium was recorded every 1 min for 10 min. The *P*_*n*_ was normalized to µmol O_2_ g^−1^ FW h^−1^.

### Chl *a* and *c* measurements

Approximately 0.2 g (fresh weight) of the discs was used for the extraction of chlorophyll (Chl) *a* and *c*. The discs were placed in 2 mL dimethyl sulfoxide for 5 min, and the absorption of the supernatant was measured at 665, 631, 582 and 480 nm using an ultraviolet absorption spectrophotometer (U-2900, HITACHI, Tokyo, Japan). Next, the same discs were placed in 3 mL acetone for 2 h. Then, the supernatant was transferred into a 10 mL tube, and 1 mL methanol and 1 mL distilled water were added. The absorbance of the supernatant was measured at 664, 631, 581 and 470 nm using an ultraviolet absorption spectrophotometer. The contents of Chl *a* and *c* were calculated according to Seely, Duncan & Vidaver (1972).

### Soluble carbohydrates and proteins

Fresh mass samples (0.1 g) were ground in 2 mL of distilled water and diluted to 10 mL after the addition of 2 mL MgCO_3_ suspension liquid. The crude mixture was centrifuged with a table centrifuge at 4,000 rpm for 5 min at 4 °C. Then, 1 mL supernatant was transferred into a glass tube and diluted to 2 mL with distilled water, and 8 mL anthrone reagent was added. The mixture was bathed in boiled water for 10 min. After the reaction medium had cooled down, the absorption at 620 nm was recorded, and the mixture was standardized with distilled water. The content of soluble carbohydrates was determined by the anthrone reagent method with glucose standards according to Tang, Gong & Chen (1985).

Fresh samples (0.1 g) were homogenized with a mortar and pestle and 5 mL of liquid nitrogen. The extract was centrifuged at 2,500 rpm for 10 min and then used to determine the content of soluble protein. The absorbance of the supernatant at 595 nm was recorded using an ultraviolet spectrophotometer. The soluble proteins of the alga was estimated using a combination of Coomassie Brilliant Blue G-250 dye and bovine albumin, as described by Kochert (1978).

### Statistical analysis

All data in the present study are reported as the mean  ± SD (*n* = 3). Prior to the analysis, tests for the normal distribution (Shapiro–Wilk test, *p* > 0.05) and homogeneity (Levene’s test, *p* > 0.05) of variance were conducted. A two-way analysis of variance (ANOVA) was used to test the effects of the nutrient and pCO_2_ levels on the RGR, net photosynthetic rate, and the contents of Chl *a* and *c* and soluble carbohydrates and protein. A Tukey HSD test was conducted to determine the significance levels of the factors (*p* < 0.05). All statistical analyses were performed using SPSS 22.0 software.

## Results

### Seawater carbonate chemistry

The effects of pCO_2_ and the nutrient level on the seawater carbonate parameters were detected (Table 1). The two-way ANOVA (*p* = 0.05) showed that pCO_2_ had a significant effect on all parameters except for TA, whereas the nutrient level did not have a significant influence on any parameter. The Tukey HSD comparison (*p* = 0.05) showed that elevated pCO_2_ decreased the pH value by 0.2 in both the enriched and non-enriched nutrient treatments and CO_3_^2 −^ by 48% (non-enriched) and 47% (enriched), but it increased the dissolved inorganic carbon (DIC) by 6% (non-enriched) and 6% (enriched), HCO_3_^−^ by 8% (non-enriched) and 8% (enriched), and CO_2_ by 122% (non-enriched) and 119% (enriched).

**Table 1:** Parameters of the seawater carbonate system in different treatments. -familz.

**Nutrient**	**pCO_2_ (µatm)**	**pH**	**DIC** (µmol kg^−1^)	**TA** (µmol kg^−1^)	**CO_3_^2-^** (µmol kg^−1^)	**HCO_3-_** (µmol kg^−1^)	**CO_2_** (µmol kg^−1^)
Non-enriched	400	8.09 ± 0.02^a^	1783.21 ± 17.17^a^	1927 ± 4.16^a^	102.16 ± 9.79^a^	1665.91 ± 24.95^a^	15.14 ± 2.01^a^
	800	7.78 ± 0.01^b^	1886.17 ± 9.29^b^	1936 ± 6.03^a^	53.57 ± 5.47^b^	1799.04 ± 13.80^b^	33.55 ± 0.90^b^
Enriched	400	8.07 ± 0.07^a^	1776.66 ± 16.03^a^	1930 ± 5.51^a^	107.71 ± 9.39^a^	1654.85 ± 23.48^a^	14.10 ± 1.92^a^
	800	7.79 ± 0.03^b^	1882.36 ± 11.88^b^	1941 ± 4.58^a^	57.60 ± 6.93^b^	1793.82 ± 17.64^b^	30.93 ± 1.17^b^

**Notes.**

DIC dissolved inorganic carbon TA total alkalinity

Data present means ± SD. Different letters indicate statistical differences (*p* < 0.05) among different experimental treatments. The unites for TA and carbonate chemistry parameters are µmol kg^−1^. Different letters indicate statistical differences (*p* < 0.05) among different experimental treatments.

### Growth

**Figure 1:**
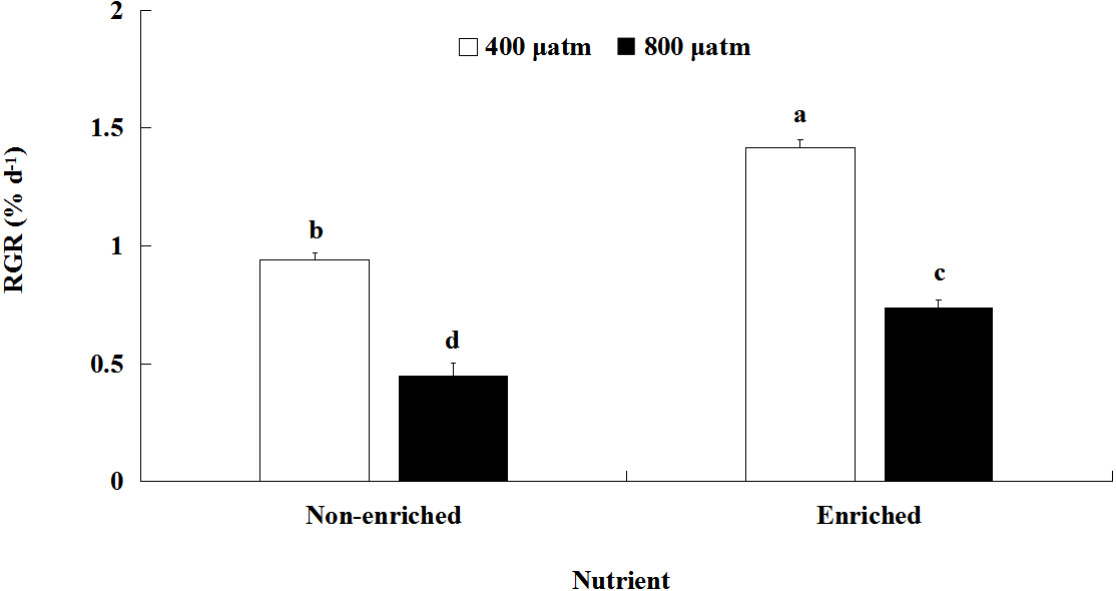
Relative growth rate (RGR) of *S. japonica* cultured for 6 days under two pCO_2_ and two nutrient levels. Data represents mean ± SD (*n* = 3 replicates). Different letters indicate statistical differences (*p* < 0.05) among different experimental treatments.

The RGR values were significantly affected by pCO_2_ and nutrients, both individually and interactively (Fig. 1; Table 2). At each pCO_2_ level, the RGR values were significantly higher under the nutrient-enriched condition than under the non-enriched condition. Similarly, for both nutrient levels, the RGR was significantly lower at the higher pCO_2_ level than at the lower level. Furthermore, the difference in RGR between the two pCO_2_ levels was further increased under nutrient-enriched conditions. The RGR showed a maximum of 1.417% day^−1^at the lower pCO_2_ level and nutrient-enriched conditions.

**Table 2:** Analysis of of two-way ANOVA showing the effects of pCO_2_ and nutrient level and their interactions on the growth (RGR), photosynthesis, chlorophyll *a* and *c* (Chl *a* and Chl *c*), soluble carbohydrates and soluble proteins.

**Parameter**	**Source of variation**	**df**	**Ms**	***F***	***p***
Relative growth rate	pCO_2_	1	1.025	687.175	<0.001
	Nutrient	1	0.436	292.409	<0.001
	pCO_2_× Nutrient	1	0.027	18.071	0.003
Net photosynthetic rate	pCO_2_	1	0.018	8.074	0.022
	Nutrient	1	0.003	1.527	0.252
	pCO_2_× Nutrient	1	0.006	2.881	0.128
Chlorophyll *a*	pCO_2_	1	0.028	55.091	<0.001
	Nutrient	1	0.005	9.81	0.014
	pCO_2_× Nutrient	1	0.009	18.293	0.003
Chlorophyll *c*	pCO_2_	1	0.001	14.502	0.005
	Nutrient	1	<0.001	0.036	0.854
	pCO_2_× Nutrient	1	<0.001	4.823	0.059
Soluble carbohydrates	pCO_2_	1	0.878	8.192	0.021
	Nutrient	1	0.519	4.844	0.059
	pCO_2_× Nutrient	1	0.549	5.119	0.054
Soluble proteins	pCO_2_	1	4.971	20.186	0.002
	Nutrient	1	9.781	39.717	<0.001
	pCO_2_× Nutrient	1	0.151	0.615	0.456

### Photosynthesis

**Figure 2:**
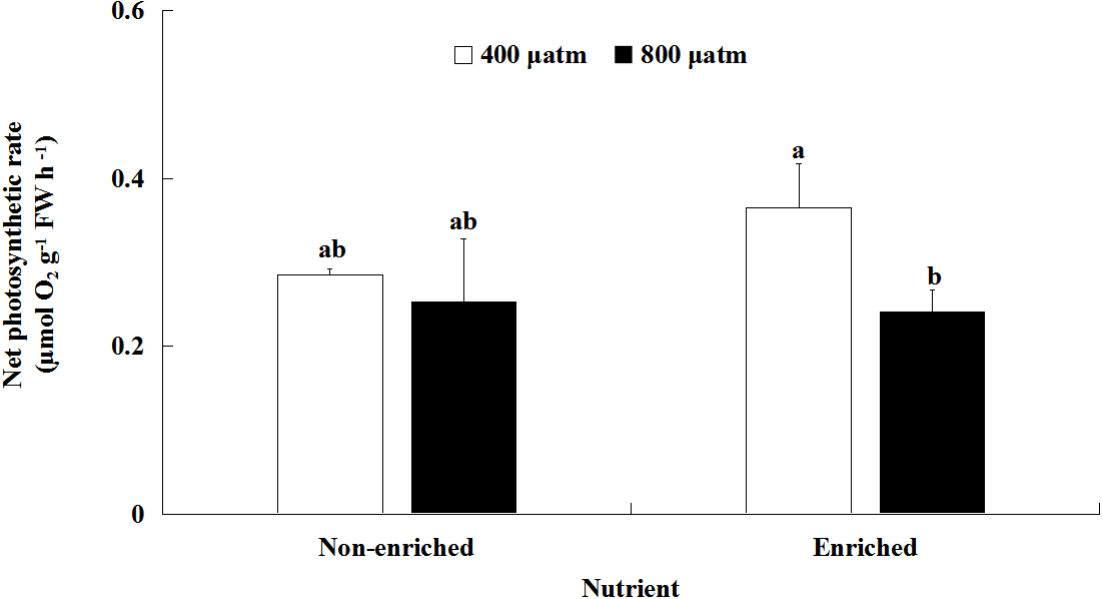
Net photosynthetic rate (*P*_*n*_) of *S. japonica* cultured for 6 days under two pCO_2_ and two nutrient levels. Data represents mean ± SD (*n* = 3 replicates). Different letters indicate statistical differences (*p* < 0.05) among different experimental treatments.

The *P*_*n*_ values were significantly different between the two pCO_2_ levels (Fig. 2; Table 2). However, there was no significant effect of nutrients on *P*_*n*_ values. A significant interaction between pCO_2_ levels and nutrients was also not detected. Under nutrient-enriched conditions, the *P*_*n*_ values were significantly higher at lower pCO_2_ than at higher pCO_2_. However, at both pCO_2_ levels, the *P*_*n*_ values did not significantly differ between the two nutrient levels. The *P*_*n*_ values showed a maximum of 0.365 µmol O_2_ g^−1^ FW h^−1^ under the lower pCO_2_ level and nutrient-enriched conditions.

### Chl *a* and *c* contents

**Figure 3:**
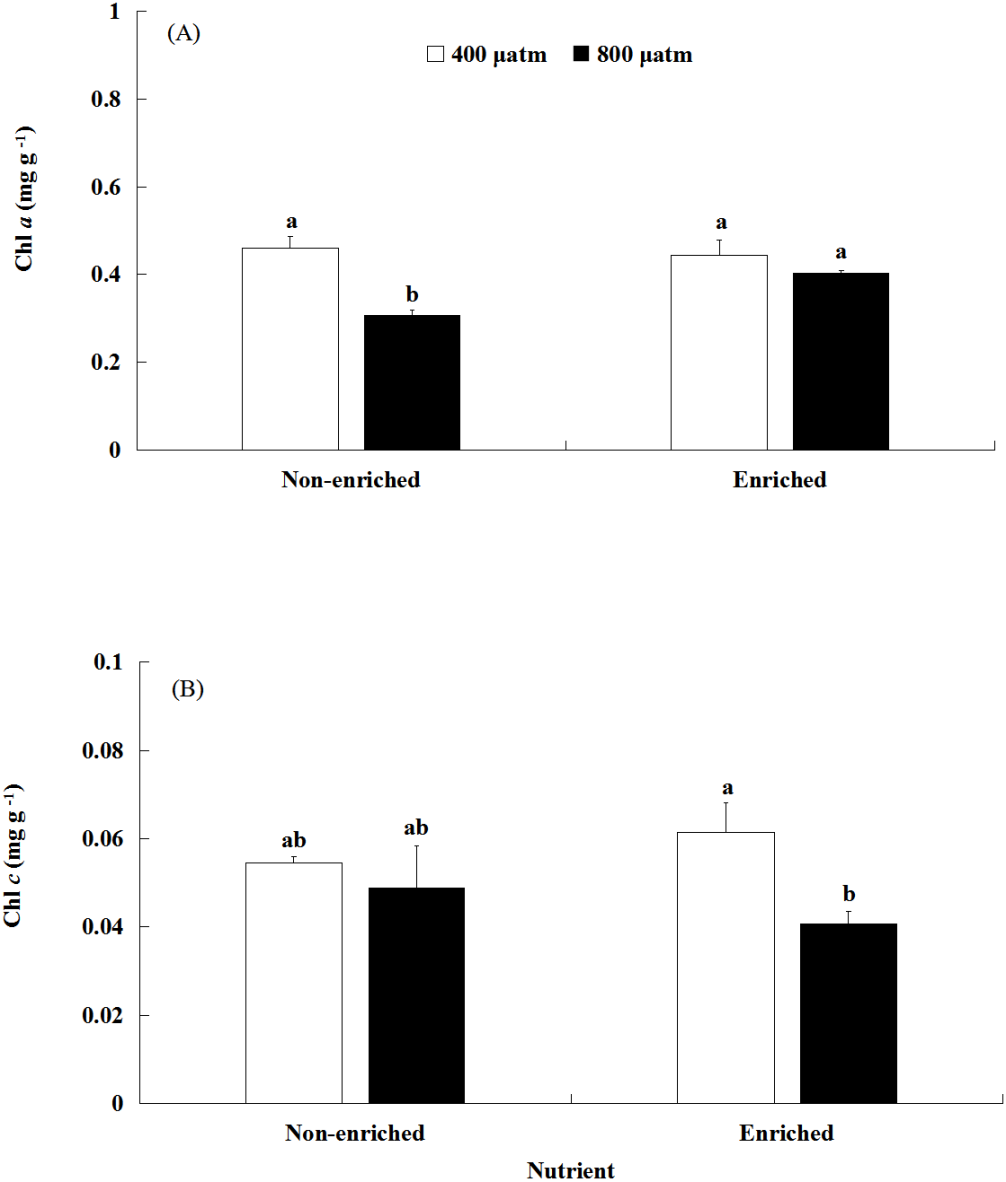
The contents of Chl *a* (A) and Chl *c* (B) of *S. japonica* cultured for 6 days under two pCO_2_ and two nutrient levels. Data represents mean ± SD (*n* = 3 replicates). Different letters indicate statistical differences (*p* < 0.05) among different experimental treatments.

The Chl *a* content was significantly affected by pCO_2_ and nutrients, both individually and interactively (Fig. 3A; Table 2). Under non-enriched conditions, the Chl *a* contents was significantly higher at lower pCO_2_ than at higher pCO_2_. However, under nutrient-enriched conditions, the Chl *a* contents showed no significant difference between the two pCO_2_ levels. Additionally, at higher pCO_2_, the Chl *a* contents was significantly higher under nutrient-enriched conditions than under non-enriched conditions. However, at lower pCO_2_, the Chl *a* content did not show significant differences between the two nutrient levels. The Chl *a* content showed a minimum of 0.308 mg g^−1^ at higher pCO_2_ and non-enriched conditions.

The Chl *c* content was significantly affected by pCO_2_ levels (Fig. 3B; Table 2). There was no significant effect of nutrients on the Chl *c* contents. Significant interaction between pCO_2_ and nutrients was not detected. Under nutrient-enriched conditions, the Chl *c* content was significantly higher at lower pCO_2_ than at higher pCO_2_. However, under non-enriched conditions, the Chl *c* content showed no significant differences between the lower pCO_2_ and higher pCO_2_ levels. The Chl *c* content showed a maximum of 0.062 mg g^−1^ at the lower pCO_2_ level and nutrient-enriched conditions.

### Soluble carbohydrates and soluble proteins

**Figure 4:**
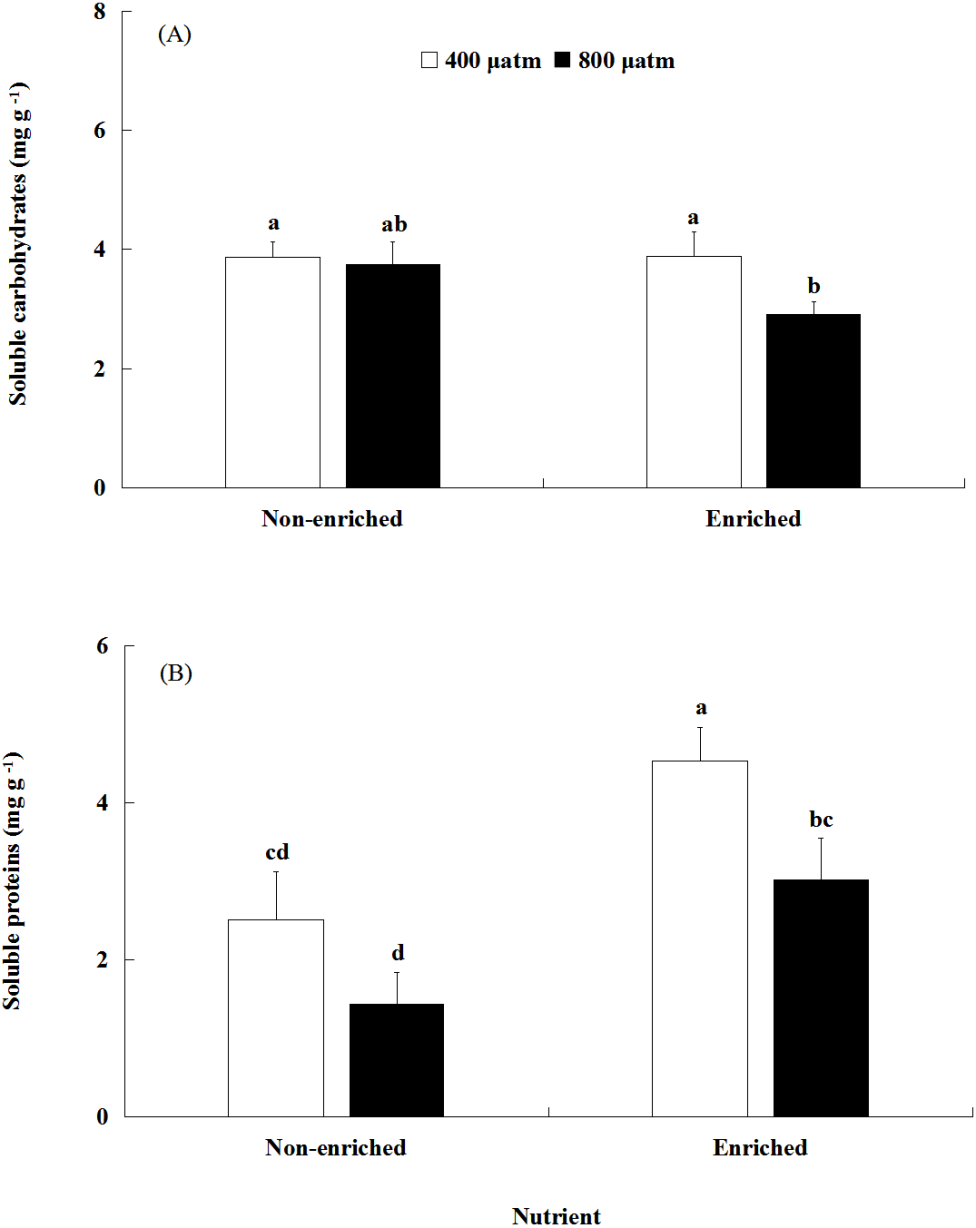
The contents of soluble carbohydrates (A) and soluble proteins (B) of *S. japonica* cultured for 6 days under two pCO_2_ and two nutrient levels. Data represents mean ± SD (*n* = 3 replicates). Different letters indicate statistical differences (*p* < 0.05) among different experimental treatments.

The soluble carbohydrates content was significantly affected by pCO_2_ (Fig. 4A, Table 2). There was no significant effect of nutrients on the soluble carbohydrates content. In addition, a significant interaction between pCO_2_ and nutrients was not detected. Under nutrient-enriched conditions, the soluble carbohydrates content at higher pCO_2_ was significantly lower than that at lower pCO_2_ levels. However, under non-enriched conditions, the soluble carbohydrates content showed no significant differences between the two pCO_2_ levels. The soluble carbohydrates content showed a minimum of 2.912 mg g^−1^ at the higher pCO_2_ level and nutrient-enriched conditions.

The soluble proteins content was significantly affected by both pCO_2_ and nutrients (Fig. 4B; Table 2). However, a significant interaction between pCO_2_ and nutrients was not detected. Under non-enriched conditions, the soluble proteins content had no significant differences between the two pCO_2_ levels. However, under nutrient-enriched conditions, the soluble proteins content at high pCO_2_ was significantly lower than that at lower pCO_2_. For both pCO_2_ levels, the soluble proteins content was significantly higher under nutrient-enriched conditions than under non-enriched conditions. The soluble proteins content showed a maximum of 4.536 mg g^−1^ at the lower pCO_2_ level and nutrient-enriched conditions.

## Discussion

In the present study, the growth of adult sporophytes of *S. japonica* was significantly decreased at the expected future pCO_2_ level of 800 µatm. Similarly, sporophytes of *Saccharina latissima* and *Fucus vesiculosus* responded to elevated pCO_2_ levels with a decrease in the growth rate (Swanson & Fox, 2007; Gutow et al., 2014). Previous studies have shown that decreased seawater pH disturbed the acid–base balance both at the cell surface and within cells (Flynn et al., 2012; Xu, Gao & Xu, 2017), thereby affecting carbonic anhydrase activity and the uptake and assimilation of nitrogen (García-Sânchez, Fernândez & Niell, 1994; Israel et al., 1999). Additionally, macroalgae could enhance the energetic cost to maintain intracellular pH stability (Xu, Gao & Xu, 2017), suggesting that the energy used for supporting growth may be reduced. These findings may partially explain why the growth of *S. japonica* decreased under OA conditions. On the other hand, the net photosynthetic rate and chlorophyll content were reduced under the high pCO_2_ levels. Similar results have been shown for *Alaria esculenta* (Gordillo et al., 2015) and *Lomentaria australis* (Van der Loos, Schmid & Leal, 2019). Elevated pCO_2_ could accelerate the degradation of chlorophyll synthesis immoderately, which is not essential for light harvesting (Gordillo et al., 1998). Lapointe & Duke (1984) also indicated that the photosynthetic activities of macroalgae were closely associated with the intracellular chlorophyll content. The reduced chlorophyll content may have contributed to a decrease in the photosynthetic rate. Additionally, according to previous study, the carotenoid contents were significantly affected by elevated pCO_2_ (Celis-Plá et al., 2017), which could influence the growth and quality of seaweed. However, in the present study, due to the limiting of experimental conditions, the concentration of carotenoid was not determined. But, we will add this parameter in future studies to investigate the effect of elevated pCO_2_ level on the growth and quality of *S. japonica*.

OA also had a negative effect on soluble carbohydrates and soluble proteins. The negative effect may be partially due to the inhibition of the enzyme activity regarding carbon assimilation due to an elevated pCO_2_ level (García-Sânchez, Fernândez & Niell, 1994), resulting in the reduction of carbohydrates synthesis (Giordano, Beardall & Raven, 2005). In *Fucus serratus* (Axelsson, Uusitalo & Ryberg, 1991), *Desmarestia aculeata* (Gordillo et al., 2016), and *Ptilota plumosa* (Gordillo et al., 2016), negative effects of OA on the soluble carbohydrates and soluble proteins were also found. The decreased soluble proteins content was likely due to a decrease in the uptake and assimilation of nitrate (Lara et al., 1987; Mercado et al., 1999), which is used for the synthesis of structural proteins involved in algal growth (Chen, Zou & Yang, 2017). On the other hand, the exposure and acclimation to elevated pCO_2_ levels would influence the reallocation of nitrogen away from Rubisco and towards other limiting components, such as non-photosynthetic processes (Bowes, 1991; Andria, Vergara & Perez-Llorens, 1999).

Significant positive effects of high nutrient availability were detected for Chl *a* content, soluble proteins content and growth. Similarly, in *U. pinnatifida* (Endo et al., 2017), the growth rate and Chl *a* content were higher in the 25% PESI-enriched treatment than in the non-enriched treatment. Stimulation of soluble proteins synthesis has also been reported in *Ulva rigida* (Gordillo, Niell & Figueroa, 2001). A higher nitrogen availability may promote the synthesis of Chl *a* and related enzymes (Dawes & Koch, 1990; Crawford, 1995). Increases in these parameters stimulated growth under high nutrient conditions and further enhanced the recruitment and natural production of kelp (Mizuta, Narumi & Yamamoto, 2001; Agatsuma et al., 2014). However, soluble carbohydrates were not significantly affected by a high nutrient supply. This result suggests that the synthesis of soluble carbohydrates was less sensitive than the other physiological parameters to changes in the nutrient supply; in future studies, the responses of multiple physiological parameters to changes in the nutrient supply should be investigated to explain these different phenomena.

Adult sporophyte growth was more severely decreased by a combination of elevated pCO_2_ and nutrient levels than by an increased pCO_2_ level individually. The results implied that natural and cultivated production of *S. japonica* will be inhibited in the future when both nutrient and pCO_2_ (800 µatm) levels are higher than the current natural seawater conditions. However, according to previous studies, the growth of adult sporophytes of *U. rigida* was reduced due to reproduction that resulted in a loss of thallus mass under high pCO_2_ and nutrient conditions (Gao et al., 2017a; Gao et al., 2017b; Gao et al., 2018b). In the present study, we did not observe a large amount of spore release. The decreased growth of adult *S. japonica* under elevated pCO_2_ and nutrient conditions may be a simple stress response to OA. In addition, high nutrient availability alleviated the negative effect of OA on the growth of *Pyropia yezoensis* (Gao et al., 2019c). The divergence of this species may be because elevated pCO_2_ induced the synthesis of functional proteins under higher nutrient conditions to scavenge reactive oxygen species and protect cells from harm caused by decreased pH (Gao et al., 2019c). According to these differential results, the interactions of elevated pCO_2_ and nutrients on seaweeds are algal species-specific. The phenomenon was possibly caused by the differences in their response mechanisms and the ability of acclimation under high pCO_2_ and nutrient conditions.

In total, climate changes present a great challenge for mariculture production of commercial macroalgae. As an environmental factor, OA could produce a negative effect on the production and quality of *S. japonica*. Continuous eutrophication aggravates deterioration, a negative effect of OA. However, due to limited physiological data in this study, more experiments regarding the responses of different developmental stages to OA and eutrophication are warranted to evaluate the development of *S. japonica* cultivation in future oceanic conditions with high pCO_2_ and nutrient levels.

## Conclusions

In this study, the combined effects of OA and eutrophication on the growth and biochemical compositions of adult sporophytes of *S. japonica* were investigated. The results showed that OA showed a significantly negative effect on growth, photosynthesis, chlorophyll, and soluble carbohydrates and proteins. Moreover, eutrophication exacerbated the negative effect of OA on growth. These results suggested that the cultivation production and commercial value of this kelp would be reduced under the future ocean conditions.

##  Supplemental Information

10.7717/peerj.8040/supp-1 Data S1 Raw data of all parameters

## Additional Information and Declarations

### Data Availability

The following information was supplied regarding data availability: The raw measurements are available in the Supplemental File.

## References

[ref-1] Agatsuma Y, Endo H, Yoshida S, Ikemori C, Takeuchi Y, Fujishima H, Nakajima K, Sano M, Kanezaki N, Imai H, Yamamoto N, Kanahama H, Matsubara T, Takahashi S, Isogai T, Taniguchi K (2014). Enhancement of *Saccharina* kelp production by nutrient supply in the Sea of Japan off southwestern Hokkaido, Japan. Journal of Applied Phycology.

[ref-2] Andria J, Vergara J, Perez-Llorens JL (1999). Biochemical responses and photosynthetic performance of *Gracilaria sp.* (Rhodophyta) from Cádiz, Spain, cultured under different inorganic carbon and nitrogen levels. European Jouranl of Phycology.

[ref-3] Axelsson L, Uusitalo J, Ryberg H, García-Reina G, Pedersén M (1991). Mechanisms for concentrating and storage of inorganic carbon in marine macroalgae. Seaweed cellular and biotechnology.

[ref-4] Bowes G (1991). Growth at elevated CO_2_: photosynthetic responses mediated through Rubisco. Plant Cell and Environment.

[ref-5] Bricker SB, Longstaff B, Dennison W, Jones A, Boicourt K, Wicks C, Woerner J (2008). Effects of nutrient enrichment in the nation’s estuaries: a decade of change. Harmful Algae.

[ref-6] Cai WJ, Hu X, Huang WJ, Murrell MC, Lehrte JC, Lohrenz SE, Chou WC, Zhai W, Hollibaugh JT, Wang Y, Zhao P, Guo X, Gundersen K, Dai M, Gong GC (2011). Acidification of subsurface coastal waters enhanced by eutrophication. Nature Geoscience.

[ref-7] Celis-Plá PSM, Martínez B, Korbee N, Hall-Spencer JM, Figueroa FL (2017). Photoprotective responses in a brown macroalgae *Cystoseira tamariscifolia* to increases in CO_2_ and temperature. Marine Environmental Research.

[ref-8] Chen B, Zou D, Yang Y (2017). Increased iron availability resulting from increased CO_2_ enhances carbon and nitrogen metabolism in the economical marine red macroalga *Pyropia haitanensis* (Rhodophyta). Chemosphere.

[ref-9] Connell SD, Russell BD (2010). The direct effects of increasing CO_2_ and temperature on noncalcifying organisms: increasing the potential for phase shifts in kelp forests. Proceeding of the Royal of Society.

[ref-10] Crawford NM (1995). Nitrate: Nutrient and signal for plant growth. The Plant Cell.

[ref-11] Dawes CJ, Koch EW (1990). Physiological responses of the red algae *Gracilaria verrucosa* and *G. tikvahiae* before and after nutrient enrichment. Bulletin of Materials Sciences.

[ref-12] Dickson AG (1990). Standard potential of the reaction: AgCl(s) + 1/2 H_2_(g) = Ag(s) + HCl(aq), and the standard acidity constant of the ion HSO_4_^−^ in synthetic seawater from 273.15 to 318.15 K. The Journal of Chemical Thermodynamics.

[ref-13] Endo H, Okumura Y, Sato Y, Agatsuma Y (2017). Interactive effects of nutrient availability, temperature, and irradiance on photosynthetic pigments and color of the brown alga *Undaria pinnatifida*. Journal of Applied Phycology.

[ref-14] Enochs IC, Manzello DP, Donham EM, Kolodziej G, Okano R, Johnston L, Young C, Iguel J, Edwards CB, Fox MD, Valentino L, Johnson S, Benavente D, Clark SJ, Carlton R, Burton T, Eynaud Y, Price NN (2015). Shift from coral to macroalgae dominance on a volcanically acidified reef. Nature Climate Change.

[ref-15] Flynn KJ, Blackford JC, Baird ME, Raven JA, Clark DR, Beardall J, Brownlee C, Fabian H, Wheeler GL (2012). Changes in pH at the exterior surface of plankton with ocean acidification. Nature Climate Change.

[ref-16] Gaitán-Espitia JD, Hancock JR, Padilla-Gamiño JL, Rivest EB, Blanchette CA, Reed DC, Hofmann GE (2014). Interactive effects of elevated temperature and pCO_2_ on early-life-history stages of the giant kelp *Macrocystis pyrifera*. Journal of Experimental Marine Biology and Ecology.

[ref-17] Gao G, Beardall J, Bao M, Wang C, Ren W, Xu J (2018a). Ocean acidification and nutrient limitation synergistically reduce growth and photosynthetic performances of a green tide alga *Ulva linza*. Biogeosciences.

[ref-18] Gao X, Choi HG, Park SK, Kim JH, Yu OH, Nam KW (2019b). Sporophytic photosynthesis and gametophytic growth of the kelp *Ecklonia stolonifera* affected by ocean acidification and warming. Aquaculture Research.

[ref-19] Gao G, Clare AS, Rose C, Caldwell GS (2017a). Intrinsic and extrinsic control of reproduction in the green tide-forming alga, *Ulva rigida*. Environmental and Experimental Botany.

[ref-20] Gao G, Clare AS, Rose C, Caldwell GS (2017b). Eutrophication and warming-driven green tides (*Ulva rigida*) are predicted to increase under future climate change scenarios. Marine Pollution Bulletin.

[ref-21] Gao G, Clare AS, Rose C, Caldwell GS (2018b). *Ulva rigida* in the future ocean: potential for carbon capture, bioremediation, and biomethane production. Global Change Biology Bioenergy.

[ref-22] Gao X, Endo H, Nahaki M, Agatsuma Y (2017c). Interactive effects of nutrient availability and temperature on growth and survival of different size classes of *Saccharina japonica* (Laminariales, Phaeophyceae). Phycologia.

[ref-23] Gao G, Gao Q, Bao M, Xu J, Li X (2019c). Nitrogen availability modulates the effects of ocean acidification on biomass yield and food quality of a marine crop, *Pyropia yezoensis*. Food Chemistry.

[ref-24] Gao X, Kim JH, Park SK, Yu OH, Kim YS, Choi HG (2019a). Diverse responses of sporophytic photochemical efficiency and gametophytic growth for two edible kelps, *Saccharina japonica* and *Undaria pinnatifida* to ocean acidification and warming. Marine Pollution Bulletin.

[ref-25] García-Sânchez MJ, Fernândez JA, Niell FX (1994). Effect of inorganic carbon supply on the photosyntetic physiology of *Gracilaria tenuistipitata*. Planta.

[ref-26] Geertz-Hansen O, Sand-Jensen K, Hansen DF, Christiansen A (1993). Growth and grazing control of abundance of the marine macroalga, *Ulva lactuca* L. in a eutrophic Danish estuary. Aquatic Botany.

[ref-27] Giordano M, Beardall J, Raven JA (2005). CO_2_ concentrating mechanisms in Algae: mechanisms, environmental modulation, and evolution. Annual Review of Plant Biology.

[ref-28] Glibert PM, Anderson DM, Gentien P, Graneli E, Sellner KG (2005). The global, complex phenomena of harmful algal blooms. Oceanography.

[ref-29] Gordillo FJL, Aguilera J, Wiencke C, Jiménez C (2015). Ocean acidification modulates the response of two Arctic kelps to ultraviolet radiation. Journal of Plant Physiology.

[ref-30] Gordillo FJL, Carmona R, Viñegla B, Wiencke C, Jiménez C (2016). Effects of simultaneous increase in temperature and ocean acidification on biochemical composition and photosynthetic performance of common macroalgae from Kongsfjorden (Svalbard). Polar Biology.

[ref-31] Gordillo FJL, Jiménez C, Figueroa FL, Niell FX (1998). Effects of increased atmospheric CO_2_ and N supply on photosynthesis, growth and cell composition of the cyanobacterium Spirulina platensis (Arthrospira). Journal of Applied Phycology.

[ref-32] Gordillo FJL, Niell FX, Figueroa FL (2001). Non-photosynthetic enhancement of growth by high CO_2_ level in the nitrophilic seaweed *Ulva rigida* C. Agardh (Chlorophyta). Planta.

[ref-33] Graham MH (2004). Effects of local deforestation on the diversity and structure of Southern California giant kelp forest food webs. Ecosystems.

[ref-34] Gutow L, Rahman MM, Bartl K, Saborowski R, Bartsch I, Wiencke C (2014). Ocean acidification affects growth but not nutritional quality of the seaweed *Fucus vesiculosus* (Phaeophyceae, Fucales). Journal of Experimental of Marine Biology and Ecology.

[ref-35] Hwang EU, Liu F, Lee KH, Ha DS, Park CS (2018). Comparison of the cultivation performance between Korean (Sugwawon No. 301) and Chinese strains (Huangguan No. 1) of kelp *Saccharina japonica* in an aquaculture farm in Korea. Algae.

[ref-36] Pachauri RK, Meyer LA, IPCC, Core Writing Team (2014). Climate change 2014: synthesis report. Contribution of working groups I, II and III to the fifth assessment report of the intergovernmental panel on climate change.

[ref-37] Israel A, Katz S, Dubinsky Z, Merrill JE, Friedlander M (1999). Photosynthetic inorganic carbon utilization and growth of *Porphyra linearis* (Rhorophyta). Journal of Applied Phycology.

[ref-38] Kochert G, Hellebust JA, Craigie JS (1978). Protein determination by dye binding. Handbook of phycological methods: physiological and biochemical methods.

[ref-39] Lapointe BE, Duke CS (1984). Biochemical strategies for growth of *Gracilaria tickvahiae* (Rhodophyta) in relation to light intensity and nitrogen availability. Journal of Phycology.

[ref-40] Lara C, Romero JM, Coronil T, Guerrero MG (1987). Interactions between photosynthetic nitrate assimilation and CO_2_ fixation in cyanobacteria. Inorganic nitrogen metabolism.

[ref-41] Lewis E, Wallace D (1998). Program developed for CO_2_ system calculations.

[ref-42] Liu F, Sun X, Wang F, Wang W, Liang Z, Lin Z, Dong Z (2014). Breeding, economic traits evaluation, and commercial cultivation of a new *Saccharina* variety “Huangguan No. 1”. Aquaculture International.

[ref-43] Liu F, Wang X, Liu J, Fu W, Duan D, Yang Y (2009). Genetic mapping of the *Laminaria japonica* (Laminariales, Phaeophyta) using amplified fragment length polymorphism markers. Journal of Phycology.

[ref-44] Liu F, Yao J, Wang X, Repnikova A, Galanin DA, Duan D (2012). Genetic diversity and structure within and between wild and cultivated *Saccharina japonica* (Laminariales, Phaeophyta) revealed by SSR markers. Aquaculture.

[ref-45] Ménesguen A, Desmit X, Dulière V, Lacroix G, Thouvenin B, Thieu V, Dussauze M (2018). How to avoid eutrophication in coastal seas? A new approach to derive river-specific combined nitrate and phosphate maximum concentrations. Science of The Total Environment.

[ref-46] Mercado J, Javier F, Gordillo L, Niell FX, Figueroa F (1999). Effects of different levels of CO_2_ on photosynthsis and cell components of the red alga *Porphyra leucosticta*. Journal of Applied Phycology.

[ref-47] Mizuta H, Maita Y (1991). Effects of nitrate supply on ammonium assimilations in the blade of *Laminaria japonica* (Phaeophyceae). Bulletin of the Faculty of Fisheries-Hokkaido University.

[ref-48] Mizuta H, Narumi H, Yamamoto H (2001). Effects of nitrate and phosphate on the growth and maturation of gametophytes of *Laminaria religiosa* Miyabe (Phaeophyceae). Suisanzoshoku.

[ref-49] Myers SS, Smith MR, Guth S, Golden CD, Vaitla B, Mueller ND, Dangour AD, Huybers P (2017). Climate change and global food systems: potential impacts on food security and undernutrition. Annual Review of Public Health.

[ref-50] Oh JC, Yu OH, Choi HG (2015). Interactive effects of increased temperature and pCO_2_ concentration on the growth of a brown algae *Ecklonia cava* in the sporophyte and gametophyte stages. Ocean and Polar Research.

[ref-51] Olischlaeger M, Bartsch I, Gutow L, Wiencke C (2012). Effects of ocean acidification on different life-cycle stages of the kelp *Laminaria hyperborea* (Phaeophyceae). Botanica Marina.

[ref-52] Ragazzola F, Foster LC, Form A, Anderson PSL, Hansteen TH, Fietzke J (2012). Ocean acidification weakens the structural integrity of coralline algae. Global Change Biology.

[ref-53] Roy RN, Roy LN, Vogel KM, Porter-Moore C, Pearson T, Good CE, Millero FJ, Campbell DM (1993). The dissociation constants of carbonic acid in seawater at salinities 5 to 45 and temperature 0 to 45 °C. Marine Chemistry.

[ref-54] Schmid R, Mills J, Dring M (1996). Influence of carbon supply on the stimulation of light-saturated photosynthesis by blue light in *Laminaria saccharina*: implications for mechanism of carbon acquisition in higher brown algae. Plant Cell Environment.

[ref-55] Seely GR, Duncan MJ, Vidaver WE (1972). Preparative and analytical extraction of pigments from brown algae with dimethyl sulfoxide. Marine Biology.

[ref-56] Selivanova ON, Zhigadlova GG, Hansen GI (2007). Revision of the systematics of algae in the order Laminariales (Phaeophyta) from the Far-Eastern Seas of Russia on the basis of molecular-phylogenetic data. Russian Journal of Marine Biology.

[ref-57] Smetacek V, Zingone A (2013). Green and golden seaweed tides on the rise. Nature.

[ref-58] Smith SV, Swaney DP, Talaue-Mcmanus L, Bartley JD, Sandhei PT, McLaughlin CJ, Dupra VC, Crossland CJ, Buddemeier RW, Maxwell BA, Wulff F (2003). Humans, hydrology, and the distribution of inorganic nutrient loading to the ocean. Bioscience.

[ref-59] Steneck RS, Graham MH, Bourque BJ, Corbett D, Erlandson JM, Estes JA, Tegner MJ (2002). Kelp forest ecosystems: biodiversity, stability, resilience and future. Environmental Conservation.

[ref-60] Swanson AK, Fox CH (2007). Altered kelp (Laminariales) phlorotannins and growth under elevated carbon dioxide and ultraviolet-B treatments can influence associated intertidal food webs. Global Change Biology.

[ref-61] Tang S, Gong M, Chen D (1985). Study of methods for determination of carbohydrates in sea water-2. The anthrone method for total amount of particulate carbohydrate. Journal of Shandong College of Oceanology.

[ref-62] Tatewaki M (1966). Formation of a crustose sporophyte with unilocular sporangia in Scitosiphon lomentaria. Phycologia.

[ref-63] Ullah H, Nagelkerken I, Goldenberg SU, Fordham DA (2018). Climate change could drive marine food web collapse through altered trophic flows and cyanobacterial proliferation. PLOS Biology.

[ref-64] Van der Loos LM, Schmid M, Leal PP (2019). Responses of macroalgae to CO_2_ enrichment cannot be inferred solely from their inorganic carbon uptake strategy. Ecology and Evolution.

[ref-65] Xiao X, Agusti S, Lin F, Li K, Pan Y, Yu Y, Zheng Y, Wu J, Duarte CM (2017). Nutrient removal from Chinese coastal waters by large-scale seaweed aquaculture. Scientific Report.

[ref-66] Xu K, Chen H, Wang W, Xu Y, Ji D, Chen C, Xie C (2017). Responses of photosynthesis and CO_2_ concentrating mechanisms of marine crop *Pyropia haitanensis* thalli to large pH variations at different time scales. Algal Research.

[ref-67] Xu Z, Gao G, Xu J (2017). Physiological response of a golden tide alga (*Sargassum muticum*) to the interaction of ocean acidification and phosphorus enrichment. Biogeosciences.

[ref-68] Xu D, Wang D, Li B, Fan X, Zhang X, Ye N, Wang Y, Mou S, Zhuang Z (2015). Effects of CO_2_ and seawater acidification on the early stages of *Saccharina japonica* development. Environmental Science & Technology.

